# Associations of sleep apnoea with glaucoma and age-related macular degeneration: an analysis in the United Kingdom Biobank and the Canadian Longitudinal Study on Aging

**DOI:** 10.1186/s12916-021-01973-y

**Published:** 2021-05-11

**Authors:** Xikun Han, Samantha Sze-Yee Lee, Nathan Ingold, Nigel McArdle, Anthony P. Khawaja, Stuart MacGregor, David A. Mackey

**Affiliations:** 1 Statistical Genetics, QIMR Berghofer Medical Research Institute, 300 Herston Road, Brisbane, Queensland 4006 Australia; 2 School of Medicine, University of Queensland, Brisbane, Australia; 3 Centre for Ophthalmology and Visual Science, Lions Eye Institute, University of Western Australia, Nedlands, Western Australia Australia; 4 School of Biomedical Science, Faculty of Health, Queensland University of Technology, Brisbane, Queensland Australia; 5 Centre for Sleep Science, School of Human Sciences, University of Western Australia, Nedlands, Australia; 6 West Australian Sleep Disorders Research Institute, Department of Pulmonary Physiology and Sleep Medicine, Sir Charles Gairdner Hospital, Nedlands, Australia; 7 National Institute for Health Research Biomedical Research Centre at Moorfields Eye Hospital NHS Foundation Trust and UCL Institute of Ophthalmology, London, UK

**Keywords:** Sleep apnoea, Glaucoma, Age-related macular degeneration, UK Biobank, CLSA, Cohort study

## Abstract

**Background:**

Sleep apnoea, a common sleep-disordered breathing condition, is characterised by upper airway collapse during sleep resulting in transient hypoxia, hypoperfusion of the optic nerve, and spike in intracranial pressure. Previous studies have reported conflicting findings on the association of sleep apnoea with glaucoma, and there are limited reports on the link between sleep apnoea and age-related macular degeneration (AMD).

**Methods:**

Middle-aged and older participants from the longitudinal United Kingdom (UK) Biobank (*n* = 502,505) and the Canadian Longitudinal Study on Aging (CLSA; *n* = 24,073) were included in this analysis. Participants in the UK Biobank and the CLSA were followed for 8 and 3 years, respectively. Participants with diagnosed glaucoma or AMD at baseline were excluded from the analysis. In the UK Biobank, sleep apnoea and incident cases of glaucoma and AMD were identified through hospital inpatient admission, primary care records, and self-reported data. Multivariable Cox proportional hazards models were used to explore associations of sleep apnoea with incidence of glaucoma or AMD.

**Results:**

During the 8-year follow-up in the UK Biobank, glaucoma incidence rates per 1000 person-years were 2.46 and 1.59 for participants with and without sleep apnoea, and the AMD incidence rates per 1000 person-years were 2.27 and 1.42 for participants with and without sleep apnoea, respectively. Multivariable adjusted hazard ratios of glaucoma and AMD risk for sleep apnoea were 1.33 (95% confidence interval [CI] 1.10–1.60, *P* = 0.003) and 1.39 (95% CI 1.15–1.68, *P* <  0.001) relative to participants without sleep apnoea. In the CLSA cohort, disease information was collected through in-person interview questionnaires. During the 3-year follow-up, glaucoma incidence rates per 1000 person-years for those with and without sleep apnoea were 9.31 and 6.97, and the AMD incidence rates per 1000 person-years were 8.44 and 6.67, respectively. In the CLSA, similar associations were identified, with glaucoma and AMD odds ratios of 1.43 (95% CI 1.13–1.79) and 1.39 (95% CI 1.08–1.77), respectively, in participants with sleep apnoea compared to those without sleep apnoea (both *P* <  0.001).

**Conclusions:**

In two large-scale prospective cohort studies, sleep apnoea is associated with a higher risk of both glaucoma and AMD. These findings indicate that patients with sleep apnoea might benefit from regular ophthalmologic examinations.

## Background

Sleep apnoea is a common sleep-disordered breathing condition that affects an estimated 17% of women and 34% of men aged 30–70 in the general population [1–3]. It is characterised by repetitive reduction or cessation of airflow in the upper airway during sleep [4] which is believed to result in transient hypoxia hypoperfusion of the optic nerve and spikes in intracranial pressure, all of which elevate the risk of optic neuropathy [5]. Indeed, higher rates of various forms of optic neuropathy, including non-arteritic anterior ischemic optic neuropathy [6, 7] and papilloedema [8], have been found in patients with sleep apnoea relative to controls.

Several previous studies have examined the link between obstructive sleep apnoea (OSA) and glaucoma, the most common cause of optic neuropathy, but findings have been inconsistent [6, 9–18]. Glaucoma itself is a leading cause of irreversible blindness worldwide [19], and the number of people with glaucoma will increase to 76 million in 2020 and to 112 million in 2040 [19, 20]. In a longitudinal study of over 7000 adults over 40 years old, Lin et al. [10] reported that those with OSA have a 5-year glaucoma hazard ratio of 1.7, adjusted for comorbidities. Meta-analyses [13–15] have similarly estimated pooled odds ratios for glaucoma of up to 2.5 in those with OSA and 5.5 in those with severe OSA. Additionally, findings from several studies have generally agreed that individuals with OSA have, on average, thinner retinal nerve fibre layers (RNFL) [2, 3, 21–23] and macular ganglion cell complex [24] compared to age-matched controls. Recently, Wozniak et al. [25] reported that the presence of OSA was independently associated with a faster rate of RNFL thinning in patients with primary open-angle glaucoma, after accounting for comorbidities. However, a number of large studies [6, 16, 18] have failed to find a significantly increased rate of glaucoma in patients with OSA. For example, Keenan et al. [18] reviewed over 3 million hospital records of patients aged 55 years and over and reported that the rate ratio for glaucoma in patients with sleep apnoea was not increased compared to the reference cohort.

Age-related macular degeneration (AMD) causes progressive visual impairment with its prevalence increasing within ageing populations [26]. Interestingly, Keenan et al. [18] found that patients with sleep apnoea had a 50% increased rate of subsequent AMD diagnosis, compared to a reference cohort. There have also been case reports [27, 28] of poorer response to anti-vascular endothelial growth factor therapy for exudative AMD in patients with sleep apnoea. However, given that only one study has reported on the possible increased incidence rates of AMD in patients with sleep apnoea, more data are required to assess this link.

Findings on the association between sleep apnoea and glaucoma have been conflicting and very few studies have examined the link between the former condition and AMD. Large-scale cohort studies would provide evidence to determine the risks of glaucoma and AMD in patients with sleep apnoea relative to participants without the condition. In this study, we investigated the associations using two large prospective cohort studies.

## Methods

### Study population

**Fig. 1:**
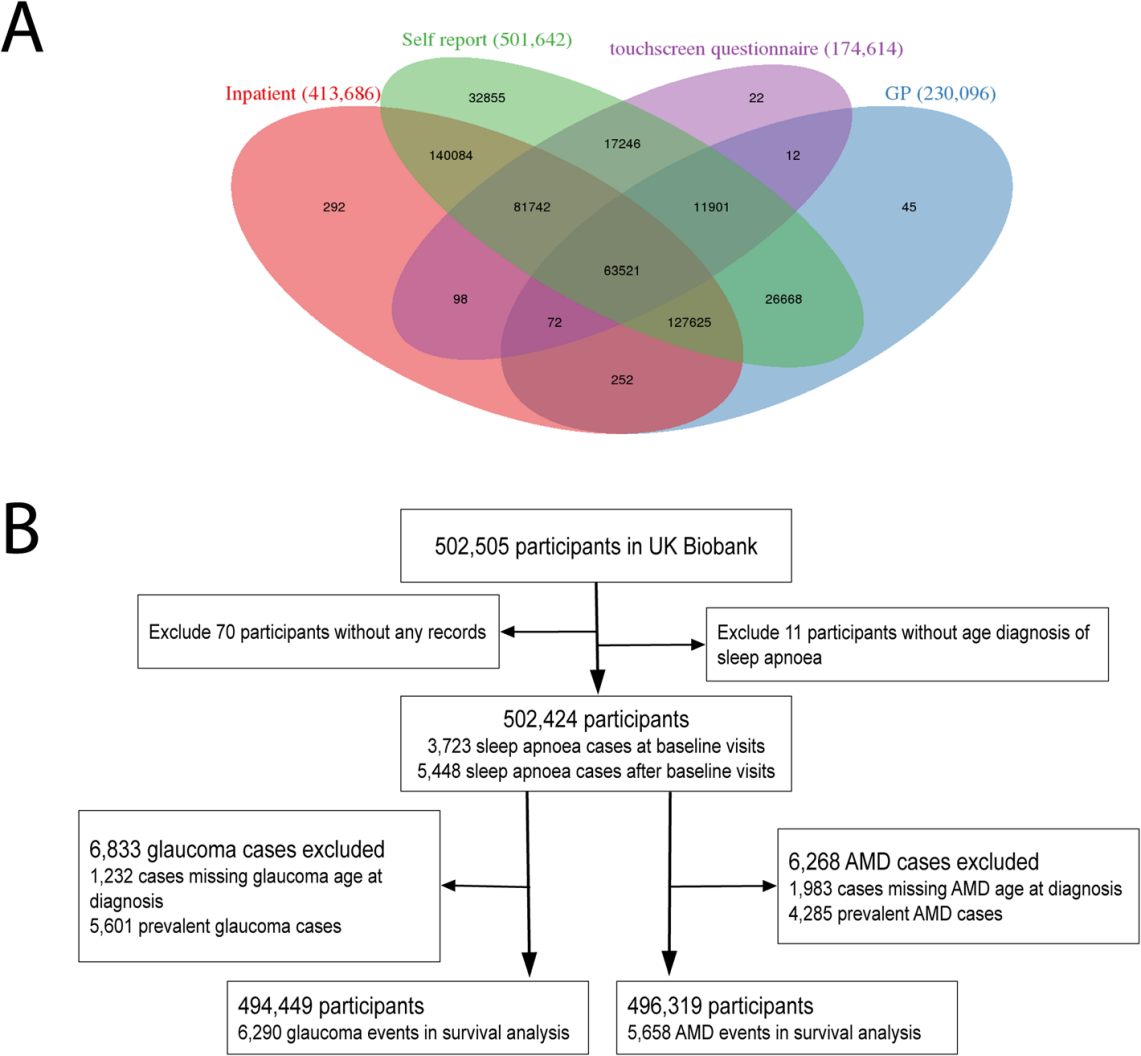
Flowchart of United Kingdom (UK) Biobank participants. A total of 502,505 participants aged between 40 and 69 at baseline were recruited between 2006 and 2010. **a** The Venn diagram of the UK Biobank participants who have any hospital inpatient records (82.3%), primary care clinical records (45.8%), giving consent to touchscreen questionnaires for self-reported eye problems/disorders (34.7%), or any records of self-reported non-cancer illness (99.8%). **b** The flowchart of the UK Biobank participants. In the analysis, glaucoma or age-related macular degeneration (AMD) cases were removed if age at diagnosis information was unavailable. Glaucoma and AMD cases were also removed if cases were prevalent before the baseline recruitment or before the age of sleep apnoea diagnosis (if sleep apnoea developed after baseline visit). The follow-up time was calculated from the time of recruitment (or age of sleep apnoea diagnosed if sleep apnoea developed after recruitment). Cox regression models were used to evaluate the associations between sleep apnoea and incidence risk of glaucoma and AMD. Sensitivity analysis was also performed to remove participants who developed sleep apnoea after the baseline visit

We used data from two prospective cohort studies: the United Kingdom (UK) Biobank study (Fig. 1) and the Canadian Longitudinal Study on Aging (CLSA; Additional file 1: Figure S1). The UK Biobank comprises over 500,000 adults, aged 40 to 69 years at the time of recruitment in 2006 to 2010 [29, 30]. The details of study design and data collection have been described previously [30]. At baseline, each participant was interviewed by trained nurses and had physical measures and biological samples taken. Health-related outcomes of the participants were collected through linkages to electronic health records, including inpatient hospital records, primary care records, and death registrations (Additional file 1: Table S1) [30].

The CLSA is a national cohort study involving 51,338 participants, aged 45 to 85 years at enrollment [31, 32]. Baseline data collection was completed in 2015, and participants will be followed up every 3 years after baseline (the first follow-up was completed in 2018). This analysed cohort included a total of 30,097 participants of their Comprehensive cohort who were interviewed in person with detailed disease information, in-depth physical assessments, and provided blood samples (Additional file 1: Table S2) [32]. Participants who were lost in the first follow-up visits, did not complete a data collection centre visit, or lacked blood samples were excluded from further analysis (Additional file 1: Figure S1 and Table S3). Finally, 24,073 participants were included in the analysis.

The UK Biobank and the CLSA study have been reviewed and approved by local research ethics boards. All participants provided informed written consent, and study procedures were performed in accordance with the World Medical Association Declaration of Helsinki ethical principles for medical research.

### Sleep apnoea, glaucoma, and AMD definitions

In the UK Biobank, sleep apnoea, glaucoma, and AMD cases were ascertained using (1) International Classification of Diseases (ICD) diagnosis codes (10th Revision, ICD-10, or 9th Revision, ICD-9), (2) record-linkage data from local general practitioners (GP), or (3) participants’ self-reported previous diagnosis (Fig. 1a). The age at diagnosis was also obtained using the three sources above. The diagnosis codes and their detailed linkage information are shown in Additional file 1: Table S1. Specifically, for sleep apnoea, the ICD-10 diagnosis code “G47.3 Sleep apnoea” was used to identify cases by linking hospital episode statistics in the UK Biobank data field 41270. The ICD-10 date of the first inpatient diagnosis was identified by linking health episode statistics (data field 41,280). Alternatively, sleep apnoea cases and recording dates were identified from primary care data, using codes that correspond to “G473”. Finally, additional self-reported sleep apnoea cases were identified from self-reported non-cancer illness (code 1123 in data field 20002), with the interpolated ages of the first diagnosis taken from data field 20009. A similar strategy was used to identify glaucoma and AMD cases. For glaucoma, we ascertained cases through (1) ICD-10 diagnosis codes H401, H408, and H409, and ICD-9 codes 3651 and 3659; (2) primary care data with the codes corresponding to the ICD10 and ICD9 glaucoma coding; (3) self-reported code 1277 in data field 20002; and (4) reported glaucoma in a survey item about eye problems or disorders (data field 6148), and the age at which glaucoma was diagnosed in data field 4689. We removed primary angle-closure glaucoma cases from all analysis. The AMD cases were identified through (1) ICD-10 diagnosis code H353 and ICD-9 code 3625, (2) primary care data with the codes corresponding to the ICD10 and ICD9 AMD coding, (3) self-reported code 1528 in data field 20002, and (4) reported AMD in a survey item about eye problems or disorders (data field 6148), and the age at which AMD was diagnosed in data field 5923.

In the CLSA, participants were interviewed in-person at data collection sites at both the baseline and the first follow-up visits (Additional file 1: Table S2) [32]. Sleep apnoea cases were defined as participants who had responded yes to the question: “Has anyone ever observed you stop breathing in your sleep” [33]. Glaucoma and AMD cases were defined by “Has a doctor ever told you that you have glaucoma?” and “Has a doctor ever told you that you have macular degeneration?”, respectively.

### Follow-up duration

In the UK Biobank, the age at earliest recorded diagnosis of glaucoma and AMD were obtained (Additional file 1: Table S1). Prevalent glaucoma or AMD cases were defined as cases whose recorded diagnosis age was before the baseline recruitment age. After the baseline visit, glaucoma or AMD cases with a recorded diagnosis age before the age of sleep apnoea diagnosis were also defined as prevalent cases. A lag period of 6 months was used for the follow-up time (that is, incident glaucoma or AMD cases should be at least 6 months after baseline recruitment or sleep apnoea diagnosis) [34]. The date of death or date of lost to follow-up, if applicable, was obtained from death registry records (data field 40000) and data field 191, respectively. At the time of analysis, the hospital admission data were available for participants up to 31 March 2017; the follow-up time was calculated from the time of recruitment (or age of sleep apnoea diagnosed if sleep apnoea developed after recruitment) to the earliest recorded age of glaucoma or AMD diagnosis, or censored at 31 March 2017 or the date of death or lost to follow-up, whichever occurred first. The follow-up duration in the CLSA is 3 years from the baseline in 2015 to the first follow-up in 2018.

### Assessment of covariates

In both the UK Biobank and CLSA, covariates were collected from standardised questionnaires, physical measurements, or biomarkers from blood assays. The detailed information for each covariate is available in Additional file 1: Tables S1 and S2. We assessed the following covariates: sex, age at recruitment, ethnicity, and smoking status (current, previous, never), waist-to-hip ratio (WHR), systolic blood pressure (SBP), high-density lipoprotein (HDL) cholesterol, total cholesterol, the presence of diabetes, and cardiovascular disease. Abdominal obesity is defined as WHR above 0.85 for women and above 0.90 for men [35]. We also included the Townsend deprivation index to represent socioeconomic status (data field 189) in the UK Biobank, with higher Townsend deprivation score representing a greater degree of deprivation [36], whereas a self-rated social standing was used in the CLSA cohort to represent socioeconomic status (the self-rated social standing ranges between 1 and 10, and higher rating means better socioeconomic status).

In the UK Biobank, cases were ascertained from different sources (Fig. 1a and Additional file 1: Table S1). We defined four indicator variables to adjust for potential selection bias (the “Statistical analysis” section below), including having any hospital inpatient records (any ICD diagnosis), having any primary care clinical records, giving consent to touchscreen questionnaires (self-reported eye problems/disorders in data field 6148), and wearing glasses. The number of participants and cases from different sources are shown in Fig. 1a and Additional file 1: Figure S2.

### Statistical analysis

The characteristics of the UK Biobank and CLSA participants were presented as mean and standard deviation (SD) for continuous variables or number and proportion for discrete variables. In the UK Biobank, Cox regression models were used to evaluate the associations between sleep apnoea and incidence of glaucoma or AMD [37]. The underlying proportional hazard assumption was evaluated by the Schoenfeld residual test [38]. Participants with glaucoma or AMD diagnosed before the index date were excluded from the survival analysis. In the basic models, we adjusted for sex, age, ethnicity, smoking status, and Townsend deprivation index. We further adjusted for WHR, SBP, diabetes, cardiovascular disease, HDL cholesterol, and total cholesterol levels. In the final model, we adjusted for indicator variables for wearing glasses, having any hospital inpatient records, having any primary care clinical records, and giving consent to touchscreen questionnaires. Missing values were imputed using imputation methods by chained equations [39]. We used the default imputation methods in R package “mice” (e.g. predictive mean matching method (“pmm”) was used for numeric variables, logistic regression imputation (“logreg”) was used for binary variables), with the default number of imputation datasets (*m* = 5). To account for the Cox model, we also imputed missing covariate values using the cumulative baseline hazard by the Nelson–Aalen estimator [40]. We additionally performed a sensitivity analysis using Cox regression models in participants without missing covariates. We also conducted another sensitivity analysis to remove participants who developed sleep apnoea after the baseline visit. We performed stratified analysis according to sex, obesity, diabetes, and cardiovascular disease status for different subgroups. For each of these variables, the potential modification effects were tested by the interaction terms with sleep apnoea status.

In the CLSA cohort, as the first follow-up was completed after 3 years of the baseline, and the specific age at diagnosis information for glaucoma and AMD was not available or incomplete, logistic regression models were used for association analysis. Similar to the analyses in the UK Biobank, we excluded participants with prevalent glaucoma or AMD cases who were reported at baseline. The above covariates were adjusted in multivariable logistic regression models, missing values were imputed using the multivariate imputation method, and different sensitivity and subgroup analyses were also performed.

All analyses were performed using R software (version 3.4.1). The *survival* package was used for Cox regression models [37]. Two-sided *P*-values less than 0.05 were considered statistically significant.

## Results

### Cohort characteristics

Of the 502,505 UK Biobank participants, 54% were women and the mean age at recruitment was 57.0 (SD 8.1) years (Table 1). The median follow-up time was 8.1 (interquartile range [IQR] 7.4 to 8.8) years. In total, there were 9182 participants with sleep apnoea, 11,926 with AMD, and 13,123 with glaucoma. Participants with sleep apnoea were more likely to be male and older, smoke, and have higher Townsend deprivation scores, higher WHR or abdominal obesity, elevated SBP, and lower HDL and total cholesterol levels. Participants with sleep apnoea also had higher rates of diabetes, cardiovascular problems, AMD, and glaucoma (Table 1). In the CLSA cohort, 24,073 participants were included in the analysis, 50% were women, and the age at baseline was 62.6 (SD 10.0) years. The median follow-up time was 3.0 (IQR 3.0 to 3.0) years. We also observed similar association patterns for sleep apnoea risk in the CLSA cohort (Table 2 and Additional file 1: Table S3).

**Table 1:** Characteristics of the UK Biobank cohort by sleep apnoea status

Variable		Sleep apnoea ***N*** = 9182	Non-sleep apnoea ***N*** = 493,231	OR (95% CI)^**2**^
Sex	Women	2636 (28.7%)	270,711 (54.9%)	1 [reference]
	Men	6546 (71.3%)	222,520 (45.1%)	3.02 (2.89–3.16)
Age at recruitment	Mean (SD), years	57.5 ± 7.6	57.0 ± 8.1	1.08 (1.06–1.11) per 10-year increase
Ethnicity	White	8529 (92.9%)	464,135 (94.1%)	1 [reference]
	Asian	241 (2.6%)	11,208 (2.3%)	1.17 (1.03–1.33)
	Black	184 (2.0%)	7873 (1.6%)	1.27 (1.09–1.47)
	Others	228 (2.5%)	10,015 (2.0%)	1.24 (1.08–1.41)
Smoking	Current	1271 (14.0%)	51,695 (10.5%)	1 [reference]
	Never	4001 (43.9%)	269,489 (55.0%)	0.60 (0.57–0.64)
	Previous	3838 (42.1%)	169,213 (34.5%)	0.92 (0.87–0.98)
Townsend deprivation score	Mean (SD)	− 0.62 ± 3.28	− 1.31 ± 3.09	1.07 (1.06–1.07)
WHR	Mean (SD)	0.95 ± 0.09	0.87 ± 0.09	2.60 (2.54–2.66) per 0.1-unit increase
Obesity	No	1841 (20.1%)	253,644 (51.4%)	1 [reference]
	Yes	7341 (79.9%)	239,587 (48.6%)	4.22 (4.01–4.45)
SBP	Mean (SD), mm Hg	139.84 ± 17.61	137.85 ± 18.70	1.06 (1.05–1.07) per 10-mmHg increase
HDL cholesterol	Mean (SD), mmol/L	1.23 ± 0.32	1.45 ± 0.38	0.14 (0.13–0.15)
Total cholesterol	Mean (SD), mmol/L	5.33 ± 1.20	5.70 ± 1.14	0.74 (0.73–0.76)
Presence of diabetes	No	7547 (82.8%)	465,900 (94.9%)	1 [reference]
	Yes	1565 (17.2%)	24,832 (5.1%)	3.89 (3.68–4.11)
Presence of cardiovascular problems	No	4201 (46.0%)	346,736 (70.6%)	1 [reference]
	Yes	4939 (54.0%)	144,361 (29.4%)	2.82 (2.71–2.94)
AMD^1^	No	8802 (95.9%)	481,685 (97.7%)	1 [reference]
	Yes	380 (4.1%)	11,546 (2.3%)	1.80 (1.62–2.00)
Glaucoma^1^	No	8756 (95.7%)	479,252 (97.4%)	1 [reference]
	Yes	390 (4.3%)	12,733 (2.6%)	1.68 (1.51–1.86)

*AMD*, age-related macular degeneration; *CI*, confidence interval; *OR*, odds ratio; *SBP*, systolic blood pressure; *SD*, standard deviation; *WHR*, waist-to-hip ratio

^1^AMD and glaucoma cases represent all prevalent and incident cases

^2^The ORs were calculated using univariate logistic regression models

**Table 2:** Characteristics of the Canadian Longitudinal Study on Aging cohort by baseline sleep apnoea status

Variable		Sleep apnoea ***N*** = 3609	Non-sleep apnoea ***N*** = 20,464	OR (95% CI)^**3**^
Sex	Women	1102 (30.5%)	10,912 (53.3%)	1 [reference]
	Men	2507 (69.5%)	9552 (46.7%)	2.60 (2.41–2.80)
Age at recruitment	Mean (SD), years	62.08 ± 9.31	62.69 ± 10.16	0.94 (0.91–0.98) per 10-year increase
Ethnicity	White	2492 (69.0%)	14,970 (73.2%)	1 [reference]
	Asian	34 (0.9%)	201 (1.0%)	1.02 (0.69–1.44)
	African	18 (0.5%)	110 (0.5%)	0.98 (0.58–1.58)
	Others	1065 (29.5%)	5183 (25.3%)	1.23 (1.14–1.33)
Smoking	Current	344 (9.5%)	1608 (7.9%)	1 [reference]
	Never	1546 (42.8%)	10,071 (49.2%)	0.72 (0.63–0.82)
	Previous	1719 (47.6%)	8785 (42.9%)	0.91 (0.81–1.04)
Social standing^1^	Mean (SD)	6.18 ± 1.93	6.27 ± 1.80	0.97 (0.95–0.99)
WHR	Mean (SD)	0.95 ± 0.09	0.90 ± 0.10	1.84 (1.77–1.92)
Obesity	No	652 (18.1%)	8065 (39.5%)	1 [reference]
	Yes	2946 (81.9%)	12,355 (60.5%)	2.95 (2.70–3.23)
SBP	Mean (SD), mm Hg	129.99 ± 18.60	127.43 ± 19.54	1.07 (1.05–1.09) per 10-mmHg increase
HDL cholesterol	Mean (SD), mmol/L	1.34 ± 0.43	1.53 ± 0.48	0.37 (0.34–0.40)
Total cholesterol	Mean (SD), mmol/L	4.92 ± 1.13	5.19 ± 1.09	0.80 (0.77–0.82)
Presence of diabetes	No	3074 (85.2%)	18,825 (92.0%)	1 [reference]
	Yes	535 (14.8%)	1639 (8.0%)	2.00 (1.80–2.22)
Presence of heart disease	No	3048 (84.6%)	18,377 (89.9%)	1 [reference]
	Yes	556 (15.4%)	2060 (10.1%)	1.63 (1.47–1.80)
Incident AMD^2^	No	3268 (97.5%)	18,783 (98.0%)	1 [reference]
	Yes	83 (2.5%)	374 (2.0%)	1.28 (1.00–1.61)
Incident glaucoma^2^	No	3312 (97.3%)	18,876 (98.0%)	1 [reference]
	Yes	93 (2.7%)	393 (2.0%)	1.35 (1.07–1.69)

*AMD*, age-related macular degeneration; *CI*, confidence interval; *OR*, odds ratio; *SBP*, systolic blood pressure; *SD*, standard deviation; *WHR*, waist-to-hip ratio

^1^The self-rated social standing ranges between 1 and 10, where higher rating means better socioeconomic status

^2^AMD and glaucoma cases represent incident cases after excluding prevalent cases

^3^The ORs were calculated using univariate logistic regression models

### Sleep apnoea and risk of incident glaucoma and AMD

In the UK Biobank analysis, we removed prevalent glaucoma or AMD cases and those without age diagnosed information (removed 6833 glaucoma and 6268 and AMD cases, Fig. 1). Over the 8-year follow-up, the incidence rates per 1000 person-years of glaucoma were 2.46 and 1.59 for participants with and without sleep apnoea, respectively. The hazard ratio (HR) of sleep apnoea on glaucoma risk was 1.68 (CI 1.40 to 2.02, *P* <  0.001) compared to participants without sleep apnoea (Table 3). There was no violation of the proportional hazards assumption. In the multivariable Cox regression model adjusting for sex, age, smoking, diabetes, cardiovascular disease, physical measurements, and cholesterol levels, the association between sleep apnoea and glaucoma attenuated slightly but remained significant (adjusted HR = 1.51, 95% CI 1.26 to 1.82, *P* <  0.001, model 4 in Table 3). To evaluate potential selection bias, we further adjusted for indicator variables for wearing glasses, having any hospital inpatient records, having any primary care clinical records, and giving consent to touchscreen questionnaires; the results were broadly similar (model 5). The associations between each of the variable and glaucoma risk from univariate and multivariable Cox regression models are presented in Additional file 1: Table S4. The association results were also essentially unchanged in sensitivity analysis without imputation of missing covariates (Additional file 1: Tables S5 and S6). We further removed participants who developed sleep apnoea after baseline visit (*N* = 5448) in the sensitivity analysis, and the results remained the same (Additional file 1: Table S7). For AMD, the incidence rates per 1000 person-years were 2.27 and 1.42 for participants with and without sleep apnoea, respectively. As with glaucoma, the conclusions were broadly the same with or without covariate adjustment (Table 3). The adjusted HR of OSA on AMD risk was 1.39 (95% confidence interval [CI] 1.15 to 1.68, *P* <  0.001).

**Table 3:** Associations of sleep apnoea with the incidence risk of glaucoma and AMD in the UK Biobank

Model^**1**^	Glaucoma event		AMD event	
	HR (95% CI)	*P* value	HR (95% CI)	*P* value
Model 1	1.68 (1.40–2.02)	< 0.001	1.74 (1.44–2.11)	< 0.001
Model 2	1.54 (1.29–1.85)	< 0.001	1.80 (1.49–2.18)	< 0.001
Model 3	1.49 (1.24–1.79)	< 0.001	1.59 (1.31–1.93)	< 0.001
Model 4	1.51 (1.26–1.82)	< 0.001	1.57 (1.30–1.90)	< 0.001
Model 5	1.33 (1.10–1.60)	0.003	1.39 (1.15–1.68)	< 0.001

*AMD*, age-related macular degeneration; *CI*, confidence interval; *HR*, hazard ratio

^1^Model 1: univariable Cox regression model; model 2: adjusted for sex and age; model 3: included model 2 variables plus Townsend deprivation index, ethnic group, smoking status, diabetes, and cardiovascular disease; model 4: included model 3 variables plus systolic blood pressure, waist-to-hip ratio, total cholesterol, and high-density lipoprotein cholesterol; model 5: included model 4 variables plus indicator variables for wearing glasses, having any hospital inpatient records, having any primary care clinical records, and giving consent to touchscreen questionnaires

**Fig. 2:**
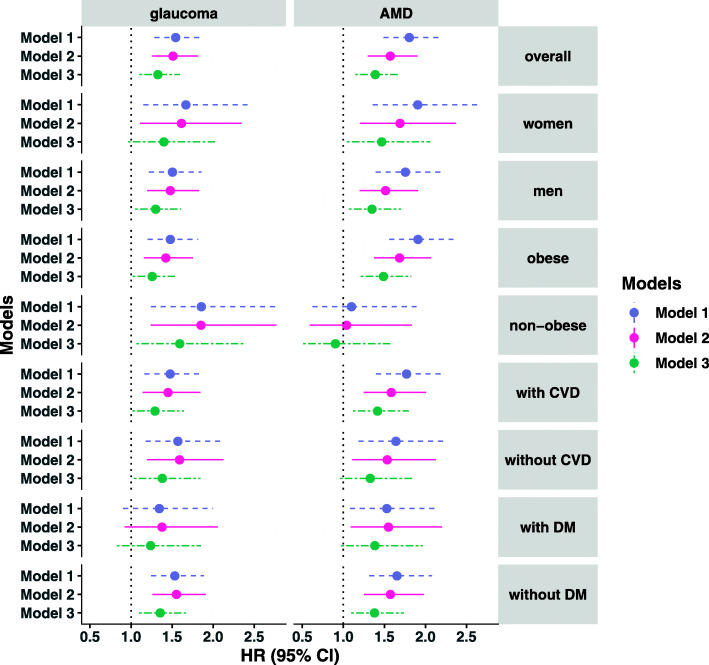
Associations of sleep apnoea with the incidence of glaucoma and AMD by different subgroups in UK Biobank. Three different models were used to adjust for covariates. Sex was not included as a covariate for sex subgroup analysis, and disease status was not included as a covariate for its specific subgroup analysis. Model 1: cox regression models adjusted for age and sex; model 2: adjusted for age, sex, Townsend deprivation index, ethnic group, smoking status, diabetes, cardiovascular disease, systolic blood pressure, waist-to-hip ratio, total cholesterol, and high-density lipoprotein cholesterol. Model 3: included model 2 variables plus indicator variables for wearing glasses, having any hospital inpatient records, having any primary care clinical records, and giving consent to touchscreen questionnaires. AMD, age-related macular degeneration; CI, confidence interval; HR, hazard ratio; DM, diabetes; CVD, cardiovascular disease

Figure 2 shows the associations of sleep apnoea with the risk of glaucoma and AMD stratified by sex, obesity, diabetes, or cardiovascular disease status, where the results were broadly consistent in different subgroups. For both glaucoma and AMD, the adjusted HR of sleep apnoea was higher in women than men. The association of sleep apnoea with the risk of AMD and glaucoma was attenuated in participants without abdominal obesity. We found no evidence of interaction effects of sleep apnoea with these subgroup factors for both glaucoma and AMD (*P*_interaction_ > 0.05 for interaction terms).

**Fig. 3:**
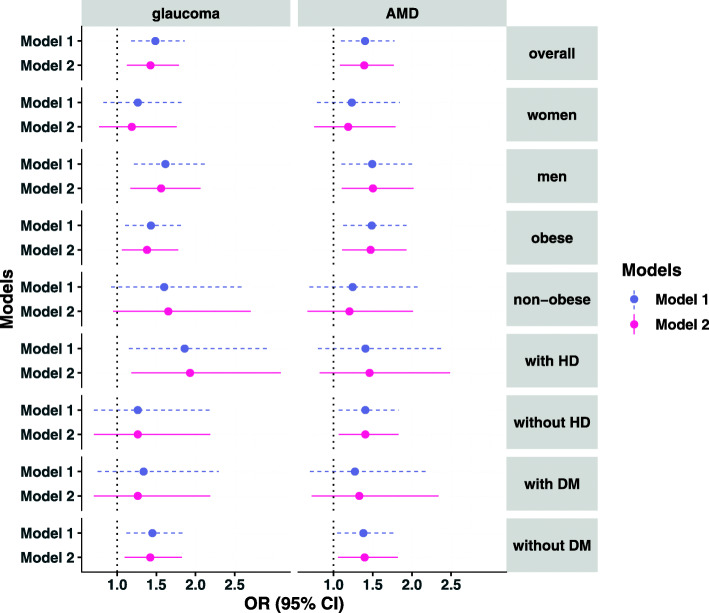
Associations of sleep apnoea with the incidence of glaucoma and AMD in the Canadian Longitudinal Study on Aging (CLSA). Three different models were used to adjust for covariates. Sex was not included as a covariate for sex subgroup analysis, and disease status was not included as a covariate for its specific subgroup analysis. Model 1: logistic regression models adjusted for age and sex; model 2: adjusted for age, sex, self-rated social standing, ethnic group, smoking status, diabetes, heart disease, systolic blood pressure, waist-to-hip ratio, total cholesterol, and high-density lipoprotein cholesterol. AMD, age-related macular degeneration; CI, confidence interval; OR, odds ratio; DM, diabetes; HD, heart disease

In the CLSA cohort, the glaucoma incidence rates per 1000 person-years were 9.31 in those with sleep apnoea and 6.97 in those without sleep apnoea, while the AMD incidence rates per 1000 person-years were 8.44 and 6.67, respectively. Similar associations with the UK Biobank cohort were found between sleep apnoea with the incident risk of glaucoma or AMD (Table 4). For instance, the multivariable-adjusted odds ratios of glaucoma and AMD risk for sleep apnoea were 1.43 (95% confidence interval [CI] 1.13 to 1.79, *P* = 0.003) and 1.39 (95% CI 1.08 to 1.77, *P* = 0.008) relative to participants without sleep apnoea (Table 4, and Additional file 1: Table S8). The results from different subgroup analysis (Fig. 3) were broadly similar, although the confidence intervals were wider compared with the UK Biobank subgroup analysis.

**Table 4:** Associations of sleep apnoea with the incidence risk of glaucoma and AMD in the Canadian Longitudinal Study on Aging (CLSA)

Model^**1**^	Incident glaucoma		Incident AMD	
	OR (95% CI)	*P* value	OR (95% CI)	*P* value
Model 1	1.38 (1.10–1.72)	0.004	1.26 (0.99–1.58)	0.06
Model 2	1.49 (1.18–1.86)	< 0.001	1.40 (1.09–1.78)	0.006
Model 3	1.44 (1.14–1.81)	0.002	1.39 (1.08–1.76)	0.009
Model 4	1.43 (1.13–1.79)	0.003	1.39 (1.08–1.77)	0.008

*AMD*, age-related macular degeneration; *CI*, confidence interval; *OR*, odds ratio

^1^Model 1: univariable logistic regression model; model 2: adjusted for sex and age; model 3: included model 2 variables plus self-rated social standing, ethnic group, smoking status, diabetes, and heart disease; model 4: included model 3 variables plus systolic blood pressure, waist-to-hip ratio, total cholesterol, and high-density lipoprotein cholesterol

## Discussion

In two large-scale cohorts of middle-aged and older adults from the UK and Canada, we found a higher incidence of glaucoma and AMD in participants with sleep apnoea relative to those without sleep apnoea. The associations were independent of age, sex, socioeconomic status, ethnic group, smoking, diabetes, cardiovascular diseases, blood pressure, and cholesterol levels.

Despite plausible mechanisms linking sleep apnoea and glaucoma, the relationship has been debated among researchers. Previous studies reporting an association between sleep apnoea and glaucoma have focused on Asian populations [10, 41]. For example, Lin et al.’s population-based matched-cohort study in Taiwan [10] reported a 5-year glaucoma HR of 1.7 in those with sleep apnoea, relative to those without sleep apnoea. This figure is not too dissimilar to the current findings of 1.3 and 1.4 in the UK and Canada, respectively. However, our results do not agree with those of Keenan et al. [18] whose study was also based in the UK but did not report a higher rate of glaucoma in those with sleep apnoea. Indeed, most studies reporting no association [16, 17] or heterogeneous results [13, 14] on the relationship between sleep apnoea and glaucoma risk had examined European or Caucasian populations. Thus, it could be possible that any association between the two conditions is more apparent in Asian populations. Unfortunately, we were unable to do further subgroup analysis for the Asian ethnic group due to the relatively small numbers of participants of Asian ancestry in the current study.

It remains curious as to why the current findings varied from those of Keenan et al. given that both studies were conducted in the UK population. A possible explanation for the difference may be the method of sampling and determining sleep apnoea, glaucoma, and AMD cases. Keenan et al. relied only on hospital records and thus may not have captured all (or even) most cases except for the most severe ones. An issue with only limiting analyses to severe cases is that it assumes that the hypothesis severity of sleep apnoea is linked with the development of glaucoma or AMD; however, it is still unclear if this is the case. The current study additionally utilised self-reported data and general practitioner record linkage, in addition to hospital-linkage records, which allowed us to include the mild and moderate cases which do not require any in-clinic procedure (e.g. sleep apnoea controlled by lifestyle modifications, medically controlled glaucoma, and non-exudative AMD). While we found a comparable glaucoma incidence to Lin et al.’s population-based matched-cohort study in Taiwan [10], the latter study reported a high incidence rate of glaucoma (per 1000 person-years of 11.26 and 6.76 for subjects with and without OSA, respectively), nearly four times that in the UK Biobank. The higher incidence rate in Lin et al.’s study may reflect the higher prevalence of glaucoma in East Asian countries (3 to 4%) compared to Europe (2.4%) [10, 19]. Moreover, Lin et al.’s cohort included a relatively large proportion of older participants (more than 70 years), which is likely to have a higher glaucoma incidence, relative to the UK Biobank (40 to 69 years) [10]. From the CLSA study, on average, the participants were 5 years older than those from the UK Biobank; indeed, the incidence rate of glaucoma was also higher in the CLSA cohort than the UK Biobank. Moreover, the prevalence of sleep apnoea in the CLSA is much higher than that in the UK Biobank, which could be due to the higher prevalence of obesity and older population in the CLSA as well as different methods of ascertaining sleep apnoea cases. However, we observed similar association patterns for different baseline characteristics with sleep apnoea in the two large biobanks (Table 1 and Table 2), suggesting they are largely comparable. Furthermore, the associations between sleep apnoea and the risk of glaucoma or AMD are similar in the two cohorts.

In our sex subgroup analysis, the association in women seemed to be attenuated in the CLSA relative to the UK Biobank. However, the confidence intervals largely overlap. Furthermore, the confidence intervals were wider in the CLSA compared with the UK Biobank, and we found no evidence of interaction effects of sleep apnoea with sex in both the UK Biobank and the CLSA. These results indicate there might be no meaningful differences for the associations between men and women between the UK Biobank and CLSA.

Instead of using the risk of incidence for glaucoma as an outcome measure, some studies used pre-clinical measures of retinal nerve fibre layer to investigate the link between OSA and optic neuropathy, and consistently found thinner retinal nerve fibre layers in middle-aged or older adults with sleep apnoea but without glaucoma [3, 23, 24, 42]. Recently, we reported that an association between thinner RNFL thickness and OSA could even be observed in young adults of European ancestry with healthy eyes [2]. In a supplementary report, Wozniak et al. [25] observed that the rate of global RNFL thinning in patients with glaucoma was on average twice as fast in those with co-existing OSA, compared to those with no OSA; hence the presence of OSA may not only be a risk factor of glaucoma, but it may also accelerate glaucoma progression.

Few studies have investigated the association between sleep apnoea and AMD. Our study revealed that sleep apnoea may be associated with an increased AMD risk, supporting Keenan et al.’s record-linkage study [18] that showed a 44% increase in AMD incidence in those with OSA, relative to a reference cohort. There is evidence that hypoxia and oxidative stress associated with OSA activates inflammatory processes [43, 44], which are believed to play a key role in the pathogenesis of AMD [45–47]. Individuals with sleep apnoea have also been observed to have thinner choroids [48], a manifestation of AMD [49], relative to controls, suggesting poorer perfusion to the outer retinal layers. Our results suggest patients with sleep apnoea might benefit from eye health screening, not only for the early detection of glaucoma but also AMD.

A main strength of the current analysis is the large scale and prospective nature of the UK Biobank and CLSA, which have detailed information on socioeconomic characteristics, physical measurements, and biomarkers from blood assays that enabled us to adjust for potential confounders in our analysis. The prospective studies also allow us to evaluate the reverse associations between glaucoma and AMD with the risk of sleep apnoea, where glaucoma or AMD status at baseline was used as the exposure, and the outcome was the incident risk of sleep apnoea during follow-up. We found no evidence of a reverse association between glaucoma and sleep apnoea risk in both UK Biobank and CLSA cohorts (Additional file 1: Tables S9 and S10), indicating the main findings that sleep apnoea is associated with a higher risk of glaucoma are unlikely to be driven by confounding factors. We observed some evidence of the reverse association between AMD and sleep apnoea risk in the UK Biobank but not in the CLSA. It is possible that there are some shared risk factors for both sleep apnoea and AMD in the UK Biobank to lead to the reverse association results, although various potential confounders were adjusted for (e.g. model 5 in Table 3). These results suggest glaucoma is not associated with the risk of sleep apnoea. However, the clinical utility of screening for sleep apnoea in patients with AMD needs further study. In our UK Biobank analysis, we used ICD codes to identify cases, which only captures patients who needed a procedure or an inpatient stay for that disorder, potentially missing cases who only had their condition diagnosed as an outpatient. To address this, we used self-reported information and record-linkage data to capture these cases. However, a limitation of self-reported data is its susceptibility to recall error, especially in age at diagnosis or misunderstanding of disease (e.g. misinterpret glaucoma as cataract). This is unlikely to affect the data significantly as the majority cases were identified from health episode statistics and general practitioner record-linkage data (Fig. 1 and Additional file 1: Figure S1). When restricting the analysis to participants having any hospital inpatient records, the association results were essentially unchanged (Additional file 1: Table S11). We further removed self-reported sleep apnoea, glaucoma, or AMD cases, and the results are essentially the same. An inherent limitation when dealing with large population-based samples is the limited ability to ascertain a high-level detail of the phenotype or disease. For instance, the detailed information for glaucoma and AMD subtypes, and continuous variables, such as apnoea-hypopnoea index, are not available, which impede further analysis for different disease subtypes and linear correlation analyses for continuous variables. Since different disease subtypes, such as low-tension glaucoma and high-tension glaucoma, may have different pathological mechanisms, their association patterns with sleep apnoea could be different [22, 50]. We did identify associations between sleep apnoea and broadly defined glaucoma and AMD, the true effect may be larger in particular subtypes. However, it would be very difficult to collect the detailed data in large-scale population-based studies such as the UK Biobank and the CLSA. A further limitation of this analysis is the lack of detailed information on treatment for sleep apnoea, such as with continuous positive airway pressure (CPAP). However, the use of CPAP is likely to ameliorate glaucoma and AMD, and it will generally bias toward the null. Moreover, a genetic study has found a high degree of homogeneity between cases from hospital records and self-reported questionnaires in the UK Biobank [51]. There is evidence of a “healthy volunteer” selection bias in the UK Biobank [52], where we observed lower incidence rates for glaucoma and AMD compared with previous reports. However, a recent study has shown that risk factor associations in the UK Biobank are broadly generalisable [53].

## Conclusion

Our results suggest that sleep apnoea is associated with an increased risk for glaucoma and AMD. It may be beneficial for individuals with sleep apnoea to undergo additional ophthalmologic examinations. Further studies are warranted to investigate the mechanisms underlying these associations.

## Supplementary Information

**Additional file 1: Figure S1.** Flowchart of Canadian Longitudinal Study on Ageing cohort participants. **Figure S2.** Venn diagram of sleep apnoea, glaucoma and age-related macular degeneration cases ascertained from different sources in UK Biobank. **Table S1.** Data coding list in UK Biobank. **Table S2.** Data coding list in the Canadian Longitudinal Study on Ageing (CLSA) cohort. **Table S3.** Characteristics of Canadian Longitudinal Study on Ageing cohort samples included in analysis versus participants excluded. **Table S4.** Univariate and multivariable regression models for the incidence risk of glaucoma and AMD in the UK Biobank. **Table S5.** Sensitivity analysis of the associations between sleep apnoea with the risk of age-related macular degeneration and glaucoma without imputation of covariates in UK Biobank. **Table S6.** The number of samples with missing covariates in UK Biobank. **Table S7.** Sensitivity analysis of the associations between sleep apnoea with the risk of age-related macular degeneration and glaucoma after removing participants who developed sleep apnoea after baseline visit in UK Biobank. **Table S8.** Univariate and multivariable regression models for the incidence risk of glaucoma and AMD in the Canadian Longitudinal Study on Aging (CLSA). **Table S9.** Reverse association analysis between age-related macular degeneration, glaucoma and the risk of sleep apnoea in UK biobank. **Table S10.** Reverse association analysis between age-related macular degeneration, glaucoma and the risk of sleep apnoea in CLSA. **Table S11.** Sensitivity analysis of the associations between sleep apnoea with the risk of age-related macular degeneration and glaucoma restricting to participants having hospital inpatient records in UK Biobank.

## Data Availability

UK Biobank data are available through the UK Biobank Access Management System https://www.ukbiobank.ac.uk/. Data are available from the Canadian Longitudinal Study on Aging (www.clsa-elcv.ca) for researchers who meet the criteria for access to de-identified CLSA data.

## Abbreviations

AMD: Age-related macular degeneration
CLSA: Canadian Longitudinal Study on Aging
ICD: International Classification of Diseases
OSA: Obstructive sleep apnoea
RNFL: Retinal nerve fibre layers
SD: Standard deviation
UKB: United Kingdom Biobank
