# Supplementary material for: Associations of sleep apnoea with glaucoma and age-related macular degeneration: an analysis in the United Kingdom Biobank and the Canadian Longitudinal Study on Aging
Source: BMC Med. 2021 May 11;19:104. doi: 10.1186/s12916-021-01973-y (PMC8111909; doi:10.1186/s12916-021-01973-y)
Supplement: Supplementary file 1 — Additional file 1: Figure S1. Flowchart of Canadian Longitudinal Study on Ageing cohort participants. Figure S2. Venn diagram of sleep apnoea, glaucoma and age-related macular degeneration cases ascertained from different sources in UK Biobank. Table S1. Data coding list in UK Biobank. Table S2. Data coding list in the Canadian Longitudinal Study on Ageing (CLSA) cohort. Table S3. Characteristics of Canadian Longitudinal Study on Ageing cohort samples included in analysis versus participants excluded. Table S4. Univariate and multivariable regression models for the incidence risk of glaucoma and AMD in the UK Biobank. Table S5. Sensitivity analysis of the associations between sleep apnoea with the risk of age-related macular degeneration and glaucoma without imputation of covariates in UK Biobank. Table S6. The number of samples with missing covariates in UK Biobank. Table S7. Sensitivity analysis of the associations between sleep apnoea with the risk of age-related macular degeneration and glaucoma after removing participants who developed sleep apnoea after baseline visit in UK Biobank. Table S8. Univariate and multivariable regression models for the incidence risk of glaucoma and AMD in the Canadian Longitudinal Study on Aging (CLSA). Table S9. Reverse association analysis between age-related macular degeneration, glaucoma and the risk of sleep apnoea in UK biobank. Table S10. Reverse association analysis between age-related macular degeneration, glaucoma and the risk of sleep apnoea in CLSA. Table S11. Sensitivity analysis of the associations between sleep apnoea with the risk of age-related macular degeneration and glaucoma restricting to participants having hospital inpatient records in UK Biobank. [file 12916_2021_1973_MOESM1_ESM.docx]

**Additional file 1: supplementary figures and tables**

**Associations of sleep apnoea with glaucoma and age-related macular degeneration: an analysis in the United Kingdom Biobank and the Canadian Longitudinal Study on Aging**

**Table of Contents:**

[Figure S1. Flowchart of Canadian Longitudinal Study on Aging cohort participants](#_ql1dxyjtu3o) **2**

[Figure S2. Venn diagram of sleep apnoea, glaucoma and age-related macular degeneration cases ascertained from different sources in UK Biobank](#_m83oxr2wp3we) **3**

[Table S1. Data coding list in UK Biobank](#_8tz4xgnzvlhw) **4**

[Table S2. Data coding list in the Canadian Longitudinal Study on Aging (CLSA) cohort](#_fqjpa0zej97c) **8**

[Table S3. Characteristics of Canadian Longitudinal Study on Aging cohort samples included in analysis versus participants excluded](#_4yxs9cb6h1yc) **9**

[Table S4. Univariate and multivariable regression models for the incidence risk of glaucoma and AMD in the UK Biobank.](#_x8siquc67wzh) **11**

[Table S5. Sensitivity analysis of the associations between sleep apnoea with the risk of age-related macular degeneration and glaucoma without imputation of covariates in UK Biobank](#_7fj42wprktli) **12**

[Table S6. The number of samples with missing covariates in UK Biobank](#_v7bmwno7elfn) **13**

[Table S7. Sensitivity analysis of the associations between sleep apnoea with the risk of age-related macular degeneration and glaucoma after removing participants who developed sleep apnoea after baseline visit in UK Biobank](#_ubbp22oe9bue) **14**

[Table S8. Univariate and multivariable regression models for the incidence risk of glaucoma and AMD in the Canadian Longitudinal Study on Aging (CLSA)](#_m3f9gsai9uio) **15**

[Table S9. Reverse association analysis between age-related macular degeneration, glaucoma and the risk of sleep apnoea in UK biobank](#_9rp4q5nicc9q) **16**

[Table S10. Reverse association analysis between age-related macular degeneration, glaucoma and the risk of sleep apnoea in CLSA](#_2a9jaemny3ud) **17**

[Table S11. Sensitivity analysis of the associations between sleep apnoea with the risk of age-related macular degeneration and glaucoma restricting to participants having hospital inpatient records in UK Biobank](#_gss8v7amyde2) **18**

####

#### Figure S1. Flowchart of Canadian Longitudinal Study on Aging cohort participants

####
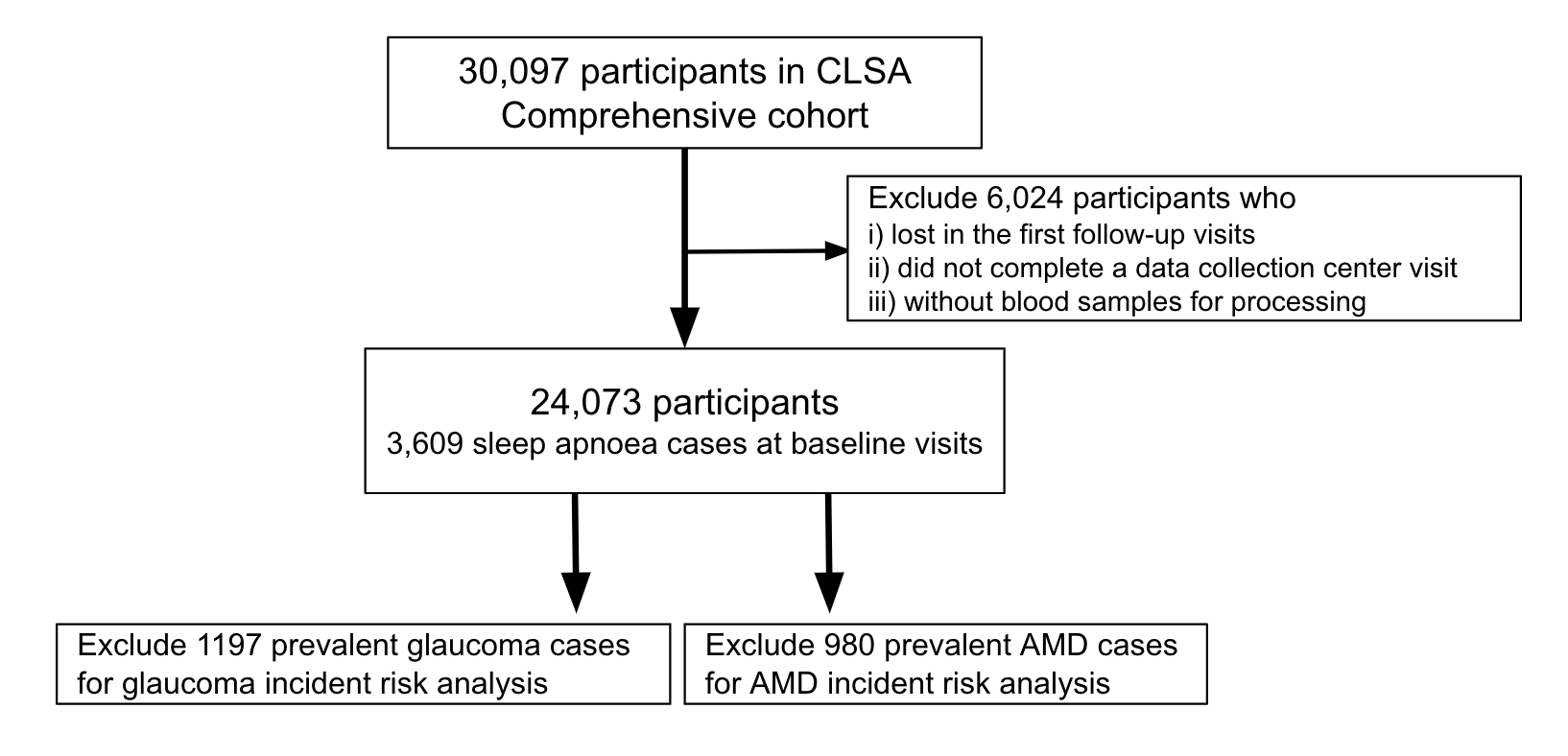


In the Canadian Longitudinal Study on Aging (CLSA) cohort, we included a Comprehensive cohort of 30,097 participants who were interviewed in person with detailed disease information, in-depth physical assessments, and provided blood samples. In the Comprehensive cohort, the baseline data collection was completed in 2015, and participants will be followed-up every 3 years after baseline (the first follow-up was completed in 2018). We excluded 6,024 participants who lost in the first follow-up visits, did not complete a data collection center visit, or without blood samples for processing from further analysis (Supplementary Table 3). Finally, 24,073 participants were included in analysis. We further excluded prevalent glaucoma or AMD cases in association analysis.

####

#### Figure S2. Venn diagram of sleep apnoea, glaucoma and age-related macular degeneration cases ascertained from different sources in UK Biobank


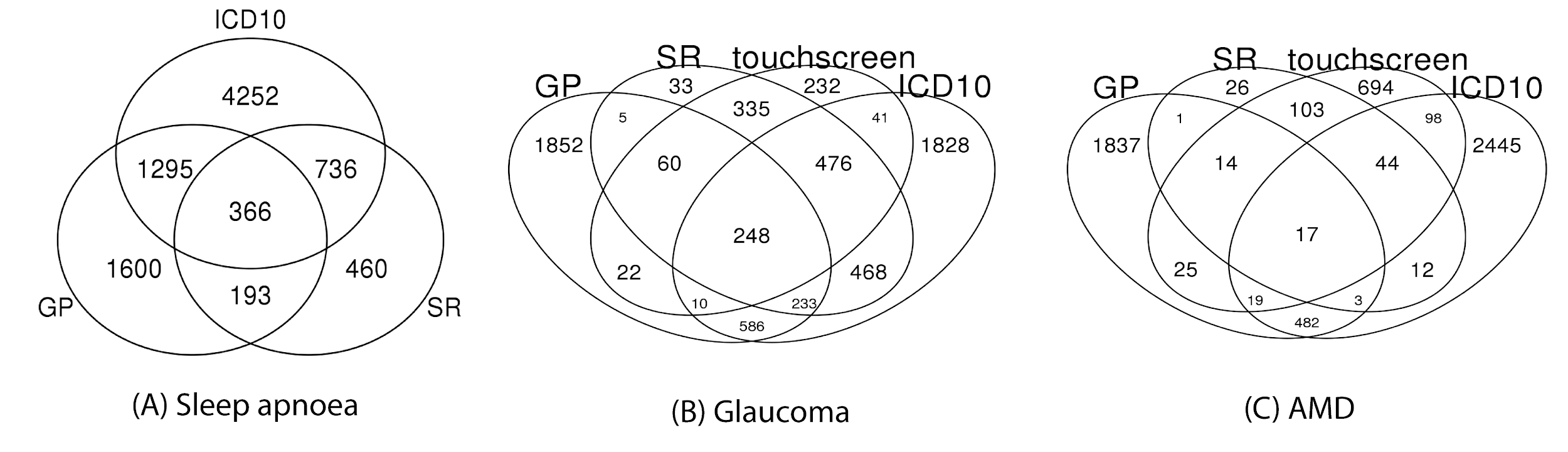


The number of cases of sleep apnoea, glaucoma and age-related macular degeneration (AMD) from the three main sources ICD10 diagnosis by linking health episode statistics, record-linkage data from local general practitioners (GP), self-reported non-cancer illness code (SR), and touchscreen questionnaires for eye problems/disorders (touchscreen). These figures show a large proportion of cases were identified from health episode statistics and record-linkage data.

####

#### Table S1. Data coding list in UK Biobank

| **Variable** | **Code_type** | **Data_field** | **Code** | **Code_description** | **Link** |
| --- | --- | --- | --- | --- | --- |
| sleep apnoea | ICD10 | 41270 | G473 | Sleep apnoea | <http://biobank.ctsu.ox.ac.uk/crystal/field.cgi?id=41270> |
| sleep apnoea | self report | 20002 | 1123 | sleep apnoea | <http://biobank.ctsu.ox.ac.uk/crystal/field.cgi?id=20002> |
| AMD | ICD10 | 41270 | H353 | Degeneration of macula and posterior pole | <http://biobank.ctsu.ox.ac.uk/crystal/field.cgi?id=41270> |
| AMD | ICD9 | 41271 | 3625 | Degeneration of macula and posterior pole | <http://biobank.ctsu.ox.ac.uk/crystal/field.cgi?id=41271> |
| AMD | self report | 20002 | 1528 | macular degeneration | <http://biobank.ctsu.ox.ac.uk/crystal/field.cgi?id=20002> |
| AMD | Eyesight | 6148 | 5 | Macular degeneration | <http://biobank.ctsu.ox.ac.uk/crystal/field.cgi?id=6148> |
| AMD | Eyesight | 5923 |  | Age macular degeneration diagnosed | <http://biobank.ctsu.ox.ac.uk/crystal/field.cgi?id=5923> |
| glaucoma | ICD10 | 41270 | H401 | Primary open-angle glaucoma | <http://biobank.ctsu.ox.ac.uk/crystal/field.cgi?id=41270> |
| glaucoma | ICD10 | 41270 | H408 | Other glaucoma |  |
| glaucoma | ICD10 | 41270 | H409 | Glaucoma, unspecified |  |
| glaucoma | ICD9 | 41271 | 3651 | Open-angle glaucoma | <http://biobank.ctsu.ox.ac.uk/crystal/field.cgi?id=41271> |
| glaucoma | ICD9 | 41271 | 3659 | Glaucoma, unspecified |  |
| glaucoma | self report | 20002 | 1277 | glaucoma | <http://biobank.ctsu.ox.ac.uk/crystal/field.cgi?id=20002> |
| glaucoma | Eyesight | 6148 |  | Glaucoma | <http://biobank.ctsu.ox.ac.uk/crystal/field.cgi?id=6148> |
| glaucoma | Eyesight | 4689 |  | Age glaucoma diagnosed | <http://biobank.ctsu.ox.ac.uk/crystal/field.cgi?id=4689> |
|  |  | 48 |  | Waist circumference | <http://biobank.ctsu.ox.ac.uk/crystal/field.cgi?id=48> |
|  |  | 49 |  | Hip circumference | <http://biobank.ctsu.ox.ac.uk/crystal/field.cgi?id=49> |
|  |  | 20116 |  | Smoking status | <http://biobank.ctsu.ox.ac.uk/crystal/field.cgi?id=20116> |
|  |  | 189 |  | Townsend deprivation index | <https://biobank.ctsu.ox.ac.uk/crystal/field.cgi?id=189> |
|  |  | 21000 |  | Ethnic background | <http://biobank.ctsu.ox.ac.uk/crystal/field.cgi?id=21000> |
|  |  | 30760 |  | HDL cholesterol | <http://biobank.ctsu.ox.ac.uk/crystal/field.cgi?id=30760> |
|  |  | 30690 |  | Total cholesterol | <http://biobank.ctsu.ox.ac.uk/crystal/field.cgi?id=30690> |
|  |  | 4080 |  | SBP, automated reading | <https://biobank.ctsu.ox.ac.uk/crystal/field.cgi?id=4080> |
|  |  | 2443 |  | Diabetes diagnosed by doctor | <http://biobank.ctsu.ox.ac.uk/crystal/field.cgi?id=2443> |
|  |  | 6150 |  | Vascular/heart problems | <http://biobank.ctsu.ox.ac.uk/crystal/field.cgi?id=6150> |
|  |  | 40000 |  | Date of death | <http://biobank.ctsu.ox.ac.uk/crystal/field.cgi?id=40000> |
|  |  | 31 |  | Sex | <http://biobank.ctsu.ox.ac.uk/crystal/field.cgi?id=31> |
|  |  | 191 |  | Date lost to follow-up | <http://biobank.ctsu.ox.ac.uk/crystal/field.cgi?id=191> |
| sleep apnoea | **Primary care^1^** | Fy03. | Fy03. |  | <http://biobank.ctsu.ox.ac.uk/crystal/label.cgi?id=3000> |
| sleep apnoea | Primary care | Fy04. | R0051 |  |  |
| sleep apnoea | Primary care | H5B.. | X0083 |  |  |
| sleep apnoea | Primary care | H5B0. | X0084 |  |  |
| sleep apnoea | Primary care | 38Da. | X0086 |  |  |
| sleep apnoea | Primary care | Q318. | X0087 |  |  |
| sleep apnoea | Primary care | R0051 | X008F |  |  |
| sleep apnoea | Primary care | R0053 | X76Gw |  |  |
| sleep apnoea | Primary care |  | X76Hk |  |  |
| sleep apnoea | Primary care |  | XE2nU |  |  |
| sleep apnoea | Primary care |  | XM08E |  |  |
| sleep apnoea | Primary care |  | R0053 |  |  |
| sleep apnoea | Primary care |  | X00pU |  |  |
| sleep apnoea | Primary care |  | X20L9 |  |  |
| sleep apnoea | Primary care |  | X20M9 |  |  |
| sleep apnoea | Primary care |  | XM0Go |  |  |
| sleep apnoea | Primary care |  | Xa08C |  |  |
| sleep apnoea | Primary care |  | XaEGP |  |  |
| sleep apnoea | Primary care |  | XaQwj |  |  |
| AMD | Primary care | F425. | F4251 |  |  |
| AMD | Primary care | F4250 | F4252 |  |  |
| AMD | Primary care | F4251 | F4256 |  |  |
| AMD | Primary care | F4252 | F4257 |  |  |
| AMD | Primary care | F4253 | F425z |  |  |
| AMD | Primary care | F4254 | F427G |  |  |
| AMD | Primary care | F4255 | X00d1 |  |  |
| AMD | Primary care | F4256 | XE15x |  |  |
| AMD | Primary care | F4257 | XE15y |  |  |
| AMD | Primary care | F425z | Xa9BN |  |  |
| AMD | Primary care | F4258 | F425. |  |  |
| AMD | Primary care | F4259 | F4250 |  |  |
| AMD | Primary care | F427G | F4254 |  |  |
| AMD | Primary care | F4343 | F4255 |  |  |
| AMD | Primary care |  | F434. |  |  |
| AMD | Primary care |  | F4343 |  |  |
| AMD | Primary care |  | X00eF |  |  |
| AMD | Primary care |  | X00eL |  |  |
| AMD | Primary care |  | X75mb |  |  |
| AMD | Primary care |  | X75mc |  |  |
| AMD | Primary care |  | X75md |  |  |
| AMD | Primary care |  | X75mg |  |  |
| AMD | Primary care |  | X75mk |  |  |
| AMD | Primary care |  | X75mp |  |  |
| AMD | Primary care |  | X75mr |  |  |
| AMD | Primary care |  | X75n2 |  |  |
| AMD | Primary care |  | X75o7 |  |  |
| AMD | Primary care |  | XE18j |  |  |
| AMD | Primary care |  | Xa9BO |  |  |
| AMD | Primary care |  | XaE0Y |  |  |
| AMD | Primary care |  | XaE0Z |  |  |
| AMD | Primary care |  | XaE0a |  |  |
| AMD | Primary care |  | XaE0b |  |  |
| AMD | Primary care |  | XaE5J |  |  |
| AMD | Primary care |  | XaE5R |  |  |
| AMD | Primary care |  | XaE5d |  |  |
| AMD | Primary care |  | XaE5t |  |  |
| AMD | Primary care |  | XaF41 |  |  |
| AMD | Primary care |  | F4253 |  |  |
| AMD | Primary care |  | X002t |  |  |
| AMD | Primary care |  | X00cx |  |  |
| AMD | Primary care |  | X00dX |  |  |
| AMD | Primary care |  | X00eJ |  |  |
| AMD | Primary care |  | X00eK |  |  |
| AMD | Primary care |  | X75nI |  |  |
| AMD | Primary care |  | X75nS |  |  |
| AMD | Primary care |  | XE0qw |  |  |
| glaucoma | Primary care | F451. | F451. |  |  |
| glaucoma | Primary care | F4510 | F4510 |  |  |
| glaucoma | Primary care | F4511 | F4511 |  |  |
| glaucoma | Primary care | F4512 | F4512 |  |  |
| glaucoma | Primary care | F4513 | F4513 |  |  |
| glaucoma | Primary care | F4515 | F4515 |  |  |
| glaucoma | Primary care | F451z | F451z |  |  |
| glaucoma | Primary care | F45y2 | F4501 |  |  |
| glaucoma | Primary care | F4501 | F4551 |  |  |
| glaucoma | Primary care | F4551 | X00ee |  |  |
| glaucoma | Primary care | F4540 | XaF9D |  |  |
| glaucoma | Primary care | F45y. | F4540 |  |  |
| glaucoma | Primary care | F45y0 | F45y. |  |  |
| glaucoma | Primary care | F45y1 | F45y0 |  |  |
| glaucoma | Primary care | F45yz | F45y1 |  |  |
| glaucoma | Primary care | FyuG0 | F45yz |  |  |
| glaucoma | Primary care | F45z. | FyuG0 |  |  |
| glaucoma | Primary care | F45.. | X00el |  |  |
| glaucoma | Primary care | F4514 | X00em |  |  |
| glaucoma | Primary care | F4544 | X00er |  |  |
| glaucoma | Primary care |  | X00et |  |  |
| glaucoma | Primary care |  | F45.. |  |  |
| glaucoma | Primary care |  | F45z. |  |  |
| glaucoma | Primary care |  | X00eg |  |  |
| glaucoma | Primary care |  | X00en |  |  |
| glaucoma | Primary care |  | X00eo |  |  |
| glaucoma | Primary care |  | X00es |  |  |
| glaucoma | Primary care |  | F4514 |  |  |
| glaucoma | Primary care |  | X00ea |  |  |

^1^ For primary care data, the two columns of coding (third and fourth columns) are version 2 (Read v2) and version 3 (CTV3 or Read v3), respectively, that correspond to their ICD10 and ICD9 codes. Some of the primary care codes in version 2 and version 3 may overlap.

####

#### Table S2. Data coding list in the Canadian Longitudinal Study on Aging (CLSA) cohort

| **Variable** | **Link** |
| --- | --- |
| Baseline sleep apnoea | <https://datapreview.clsa-elcv.ca/mica/variable/com:SNO_STOPBREATH_MCQ:Collected#/> |
| Baseline AMD | <https://datapreview.clsa-elcv.ca/mica/variable/com:SNO_STOPBREATH_MCQ:Collected#/> |
| AMD | <https://datapreview.clsa-elcv.ca/mica/variable/cof1%3ACCC_MACDEG_COF1%3ACollected#/> |
| Baseline glaucoma | <https://datapreview.clsa-elcv.ca/mica/variable/com%3AVIS_GLAUC_COM%3ACollected> |
| Follow-up glaucoma | <https://datapreview.clsa-elcv.ca/mica/variable/cof1%3AICQ_GLAUC_COF1%3ACollected> |
| Sex | <https://datapreview.clsa-elcv.ca/mica/variable/com%3ASEX_ASK_COM%3ACollected#/> |
| Baseline age | <https://datapreview.clsa-elcv.ca/mica/variable/com%3AAGE_NMBR_COM%3ACollected> |
| Age at follow-up | <https://datapreview.clsa-elcv.ca/mica/variable/cof1%3AAGE_NMBR_COF1%3ACollected> |
| Ethnicity | <https://datapreview.clsa-elcv.ca/mica/variable/com%3ASPR_OUTPUT_ETHN_COM%3ACollected#/> |
| Smoking status | <https://datapreview.clsa-elcv.ca/mica/variable/com%3AICQ_SMOKE_COM%3ACollected#/> |
| Self-rated social standing | <https://datapreview.clsa-elcv.ca/mica/variable/com%3ASEQ_LADDER_MCQ%3ACollected#/> |
| Waist circumference | <https://datapreview.clsa-elcv.ca/mica/variable/com%3AWHC_WAIST_CM_COM%3ACollected#/> |
| Hips circumference | <https://datapreview.clsa-elcv.ca/mica/variable/com%3AWHC_HIP_CM_COM%3ACollected#/> |
| Diabetes | <https://datapreview.clsa-elcv.ca/mica/variable/com%3ADIA_TYPE_COM%3ACollected#/> |
| Heart disease | <https://datapreview.clsa-elcv.ca/mica/variable/com%3ACCC_HEART_COM%3ACollected#/> |
| Systolic blood pressure | <https://datapreview.clsa-elcv.ca/mica/variable/com%3ACCC_HEART_COM%3ACollected#/> |
| High-Density Lipoprotein | <https://datapreview.clsa-elcv.ca/mica/variable/com%3ABLD_HDL_COM%3ACollected> |
| Cholesterol | <https://datapreview.clsa-elcv.ca/mica/variable/com%3ABLD_CHOL_COM%3ACollected> |

####

#### Table S3. Characteristics of Canadian Longitudinal Study on Aging cohort samples included in analysis versus participants excluded

| **Variable** |  | **Included**  **N = 24,073** | **Not included**  **N = 6,024** | **P value** |
| --- | --- | --- | --- | --- |
| Sex | Women | 12014 (49.9%) | 3306 (54.9%) | < 0.001 |
|  | Men | 12059 (50.1%) | 2718 (45.1%) |  |
| Age at recruitment | mean (SD), years | 62.6 ± 10.0 | 64.4 ± 10.9 | < 0.001 |
| Ethnic | White | 17462 (72.5%) | 3843 (63.8%) | < 0.001 |
|  | Asian | 235 (1.0%) | 69 (1.1%) |  |
|  | African | 128 (0.5%) | 53 (0.9%) |  |
|  | Other | 6248 (26.0%) | 2059 (34.2%) |  |
| Smoking | current | 1952 (8.1%) | 758 (12.6%) | < 0.001 |
|  | never | 11617 (48.3%) | 2626 (43.6%) |  |
|  | previous | 10504 (43.6%) | 2640 (43.8%) |  |
| Social standing^1^ | mean (SD) | 6.26 ± 1.82 | 6.28 ± 1.77 | 0.44 |
| WHR | mean (SD) | 0.90 ± 0.10 | 0.90 ± 0.10 | 0.40 |
| Obesity | No | 8717 (36.3%) | 2075 (35.5%) | **0.27** |
|  | Yes | 15301 (63.7%) | 3769 (64.5%) |  |
| SBP | mean (SD), mm Hg | 127.81 ± 19.43 | 129.68 ± 20.79 | < 0.001 |
| HDL-cholesterol | mean (SD), mmol/L | 1.50 ± 0.48 | 1.46 ± 0.47 | < 0.001 |
| Total cholesterol | mean (SD), mmol/L | 5.15 ± 1.10 | 5.06 ± 1.18 | < 0.001 |
| Presence of diabetes | No | 21899 (91.0%) | 5241 (87%) | < 0.001 |
|  | Yes | 2174 (9.0%) | 783 (13%) |  |
| Presence of heart disease | No | 21425 (89.1%) | 5108 (85.2%) | < 0.001 |
|  | Yes | 2616 (10.9%) | 887 (14.8%) |  |
| Baseline sleep apnoea | No | 20464 (85.0%) | 3490 (84.6%) | **0.50** |
|  | Yes | 3609 (15.0%) | 636 (15.4%) |  |
| Baseline AMD | No | 22966 (96.0%) | 5637 (94.8%) | < 0.001 |
|  | Yes | 968 (4.0%) | 312 (5.2%) |  |
| Follow-up AMD | No | 22317 (94.7%) | 2910 (94.7%) | **1** |
|  | Yes | 1247 (5.3%) | 162 (5.3%) |  |
| Baseline glaucoma | No | 22784 (95.0%) | 5624 (94.4%) | 0.04 |
|  | Yes | 1189 (5.0%) | 336 (5.6%) |  |
| Follow-up glaucoma | No | 5624 (94.4%) | 2932 (93.8%) | **0.35** |
|  | Yes | 336 (5.6%) | 194 (6.2%) |  |

AMD, age-related macular degeneration; CI, confidence interval; OR, odds ratio; SBP, systolic blood pressure; SD, standard deviation; WHR, waist-to-hip ratio.

^1^The self-rated social standing ranges between 1 to 10, where higher rating means better socioeconomic status.

^2^AMD cases and glaucoma cases represent incident cases after excluding all prevalent cases.

^3^ Sleep apnoea cases were defined as “Stopped breathing in sleep” in Canadian Longitudinal Study on Aging.

^4^The ORs were calculated using univariate logistic regression models.

In the CLSA cohort, 6,024 participants who lost in the first follow-up visits, did not complete a data collection center visit, or without blood samples for processing were excluded from further analysis. This table shows that participants excluded from the CLSA cohort were more likely to be female, older, smoker, having diabetes and heart diseases. However, some key variables, such as obesity, baseline sleep apnoea, glaucoma and AMD status in the follow-up visit, are balanced between participants included versus excluded.

####

####

#### Table S4. Univariate and multivariable regression models for the incidence risk of glaucoma and AMD in the UK Biobank.

| **Variables^1^** | **Glaucoma risk^2^** | |  | **AMD risk** | |
| --- | --- | --- | --- | --- | --- |
|  | Univariate | Multivariable |  | Univariate | Multivariable |
| Sleep apnoea | 1.68 (1.40-2.02) | 1.33 (1.10-1.60) |  | 1.74 (1.44-2.11) | 1.39 (1.15-1.68) |
| Age at recruitment,  per 10-year increase | 2.47 (2.38-2.57) | 2.19 (2.10-2.28) |  | 3.17 (3.03-3.31) | 2.84 (2.71-2.97) |
| Sex (Men vs Women) | 1.24 (1.18-1.30) | 1.35 (1.26-1.44) |  | 0.84 (0.80-0.89) | 0.75 (0.70-0.80) |
| Ethnic (Asian vs White) | 1.12 (0.95-1.32) | 1.19 (1.01-1.41) |  | 1.16 (0.99-1.37) | 1.38 (1.16-1.63) |
| Ethnic (Black) | 1.92 (1.65-2.23) | 2.41 (2.06-2.82) |  | 0.68 (0.53-0.88) | 0.95 (0.73-1.24) |
| Ethnic (Other) | 0.94 (0.78-1.13) | 1.15 (0.95-1.40) |  | 0.83 (0.68-1.02) | 1.13 (0.91-1.39) |
| Smoking(never vs current) | 1.15 (1.05-1.26) | 1.07 (0.98-1.17) |  | 1.07 (0.97-1.18) | 0.94 (0.85-1.03) |
| Smoking(previous) | 1.28 (1.17-1.41) | 0.98 (0.90-1.08) |  | 1.40 (1.27-1.54) | 1.01 (0.92-1.11) |
| Townsend deprivation score | 1.00 (0.99-1.01) | 1.00 (0.99-1.01) |  | 1.00 (0.99-1.01) | 1.00 (0.99-1.01) |
| WHR, per 0.1 unit change | 1.19 (1.16-1.22) | 0.96 (0.93-1.00) |  | 1.12 (1.09-1.16) | 1.03 (0.99-1.08) |
| SBP, per 10 mmHg increase | 1.13 (1.11-1.14) | 1.02 (1.00-1.03) |  | 1.13 (1.12-1.15) | 1.01 (1.00-1.03) |
| HDL-cholesterol | 1.03 (0.96-1.10) | 1.11 (1.03-1.20) |  | 1.08 (1.01-1.15) | 1.07 (0.98-1.16) |
| Total cholesterol | 0.99 (0.97-1.02) | 1.03 (1.00-1.05) |  | 0.96 (0.94-0.98) | 0.98 (0.96-1.01) |
| Presence of diabetes | 1.71 (1.56-1.87) | 1.21 (1.09-1.33) |  | 2.42 (2.23-2.63) | 1.66 (1.51-1.82) |
| Presence of cardiovascular problems | 1.52 (1.45-1.60) | 1.00 (0.95-1.06) |  | 1.77 (1.68-1.87) | 1.08 (1.02-1.14) |
| Wearing glasses | 3.13 (2.76-3.55) | 1.73 (1.53-1.97) |  | 3.01 (2.65-3.43) | 1.26 (1.11-1.44) |
| Having any hospital inpatient records | 3.02 (2.74-3.33) | 2.35 (2.13-2.60) |  | 4.01 (3.57-4.51) | 2.81 (2.50-3.17) |
| Having any primary care clinical records | 1.72 (1.63-1.81) | 1.80 (1.71-1.90) |  | 1.98 (1.88-2.09) | 2.02 (1.91-2.13) |
| Giving consent to touchscreen questionnaires | 2.80 (2.66-2.94) | 2.61 (2.48-2.75) |  | 1.70 (1.61-1.79) | 1.54 (1.46-1.62) |

AMD, age-related macular degeneration; CI, confidence interval; SBP, systolic blood pressure; WHR, waist-to-hip ratio.

^1^  The unit for each variable is the same as Table 1 if unspecified.

^2^ The hazard ratios and 95% confidence intervals were calculated from univariate and multivariable cox regression models.

#### Table S5. Sensitivity analysis of the associations between sleep apnoea with the risk of age-related macular degeneration and glaucoma without imputation of covariates in UK Biobank

| **Model^1^** | **Glaucoma event** | |  | **AMD event** | |
| --- | --- | --- | --- | --- | --- |
|  | HR (95% CI) | P value |  | HR (95% CI) | P value |
| Model 1 | 1.68 (1.40-2.02) | < 0.001 |  | 1.74 (1.44-2.11) | < 0.001 |
| Model 2 | 1.54 (1.29-1.85) | < 0.001 |  | 1.80 (1.49-2.18) | < 0.001 |
| Model 3 | 1.49 (1.24-1.80) | < 0.001 |  | 1.59 (1.32-1.93) | < 0.001 |
| Model 4 | 1.59 (1.31-1.93) | < 0.001 |  | 1.56 (1.27-1.92) | < 0.001 |
| Model 5 | 1.40 (1.15-1.70) | < 0.001 |  | 1.38 (1.12-1.70) | 0.002 |

AMD, age-related macular degeneration; CI, confidence interval; HR, hazard ratio;

^1^ Model 1: univariable Cox regression model; Model 2: adjusted for sex and age; Model 3: included model 2 variables plus Townsend deprivation index, ethnic group, smoking status, diabetes, and cardiovascular disease ; Model 4: included model 3 variables plus systolic blood pressure, waist-to-hip ratio, total cholesterol, and high-density lipoprotein cholesterol; Model 5: included model 4 variables plus indicator variables for wearing glasses, having any hospital inpatient records, having any primary care clinical records, giving consent to touchscreen questionnaire.

#### Table S6. The number of samples with missing covariates in UK Biobank

| **Variable** | **Number** | **Proportion^1^** |
| --- | --- | --- |
| Townsend deprivation score | 624 | 0.0012 |
| HDL-cholesterol | 70372 | 0.14 |
| Systolic blood pressure | 1326 | 0.0026 |
| Waist-to-hip ratio | 2205 | 0.0044 |
| Total cholesterol | 31661 | 0.063 |

^1^ The proportions were calculated from 502,505 UK Biobank participants.

####

#### Table S7. Sensitivity analysis of the associations between sleep apnoea with the risk of age-related macular degeneration and glaucoma after removing participants who developed sleep apnoea after baseline visit in UK Biobank

| **Model^1^** | **Glaucoma event** | |  | **AMD event** | |
| --- | --- | --- | --- | --- | --- |
|  | HR (95% CI) | P value |  | HR (95% CI) | P value |
| Model 1 | 1.83 (1.47-2.28) | < 0.001 |  | 1.71 (1.35-2.17) | < 0.001 |
| Model 2 | 1.61 (1.29-2.00) | < 0.001 |  | 1.68 (1.32-2.13) | < 0.001 |
| Model 3 | 1.55 (1.25-1.93) | < 0.001 |  | 1.48 (1.16-1.87) | 0.001 |
| Model 4 | 1.57 (1.26-1.96) | < 0.001 |  | 1.46 (1.15-1.86) | 0.002 |
| Model 5 | 1.37 (1.10-1.71) | 0.005 |  | 1.29 (1.02-1.64) | 0.04 |

CI, confidence interval; HR, hazard ratio;

^1^ Model 1: univariable Cox regression model; Model 2: adjusted for sex and age; Model 3: included model 2 variables plus Townsend deprivation index, ethnic group, smoking status, diabetes, and cardiovascular disease ; Model 4: included model 3 variables plus systolic blood pressure, waist-to-hip ratio, total cholesterol, and high-density lipoprotein cholesterol; Model 5: included model 4 variables plus indicator variables for wearing glasses, having any hospital inpatient records, having any primary care clinical records, giving consent to touchscreen questionnaires.

#### Table S8. Univariate and multivariable regression models for the incidence risk of glaucoma and AMD in the Canadian Longitudinal Study on Aging (CLSA)

| **Variables^1^** | **Glaucoma risk^2^** | |  | **AMD risk** | |
| --- | --- | --- | --- | --- | --- |
|  | Univariate | Multivariable |  | Univariate | Multivariable |
| Sleep apnoea | 1.38 (1.10-1.72) | 1.43 (1.13-1.79) |  | 1.26 (0.99-1.58) | 1.39 (1.08-1.77) |
| Age at recruitment,  per 10-year increase | 1.83 (1.68-2.01) | 1.86 (1.69-2.05) |  | 2.16 (1.97-2.38) | 2.20 (1.98-2.44) |
| Sex (Men vs Women) | 1.02 (0.86-1.22) | 0.80 (0.62-1.05) |  | 0.97 (0.81-1.16) | 0.94 (0.71-1.23) |
| Ethnic (Asian vs White) | 0.85 (0.26-2.02) | 0.98 (0.30-2.34) |  | 1.78 (0.80-3.40) | 2.06 (0.91-4.00) |
| Ethnic (African) | 0.78 (0.13-2.45) | 0.79 (0.13-2.52) |  | 0.41 (0.02-1.83) | 0.42 (0.02-1.92) |
| Ethnic (Other) | 1.19 (0.97-1.44) | 0.98 (0.80-1.19) |  | 1.05 (0.85-1.29) | 0.84 (0.68-1.04) |
| Smoking(never vs current) | 0.92 (0.66-1.31) | 0.80 (0.57-1.14) |  | 1.20 (0.84-1.78) | 0.91 (0.63-1.36) |
| Smoking(previous) | 1.13 (0.82-1.61) | 0.83 (0.59-1.19) |  | 1.27 (0.89-1.88) | 0.82 (0.57-1.22) |
| Social standing | 0.95 (0.91-1.00) | 0.96 (0.92-1.01) |  | 0.99 (0.95-1.05) | 1.01 (0.96-1.06) |
| WHR, per 0.1 unit change | 1.14 (1.04-1.25) | 1.14 (0.98-1.31) |  | 1.05 (0.96-1.16) | 0.96 (0.82-1.12) |
| SBP, per 10 mmHg increase | 1.06 (1.01-1.10) | 0.96 (0.92-1.01) |  | 1.12 (1.07-1.17) | 1.01 (0.96-1.06) |
| HDL-cholesterol | 0.94 (0.78-1.13) | 0.99 (0.79-1.23) |  | 1.01 (0.83-1.22) | 0.97 (0.77-1.21) |
| Total cholesterol | 0.91 (0.84-0.99) | 1.04 (0.95-1.14) |  | 0.89 (0.82-0.97) | 1.02 (0.93-1.12) |
| Presence of diabetes | 1.61 (1.22-2.07) | 1.28 (0.96-1.68) |  | 1.74 (1.33-2.24) | 1.42 (1.07-1.88) |
| Presence of heart disease | 1.84 (1.45-2.31) | 1.20 (0.93-1.53) |  | 1.70 (1.32-2.16) | 1.04 (0.80-1.35) |

AMD, age-related macular degeneration; CI, confidence interval; SBP, systolic blood pressure; WHR, waist-to-hip ratio.

^1^  The unit for each variable is the same as Table 2 if unspecified.

^2^ The odds ratios and 95% confidence intervals were calculated from univariate and multivariable logistics regression models.

#### Table S9. Reverse association analysis between age-related macular degeneration, glaucoma and the risk of sleep apnoea in UK biobank

| **Model^1^** | **Glaucoma as exposure** | |  | **AMD as exposure** | |
| --- | --- | --- | --- | --- | --- |
|  | HR (95% CI) | P value |  | HR (95% CI) | P value |
| Model 1 | 1.48 (1.23-1.79 | < 0.001 |  | 2.14 (1.79-2.56) | < 0.001 |
| Model 2 | 1.40 (1.16-1.69) | < 0.001 |  | 2.28 (1.90-2.73) | < 0.001 |
| Model 3 | 1.25 (1.03-1.51) | 0.02 |  | 1.88 (1.57-2.25) | < 0.001 |
| Model 4 | 1.24 (1.03-1.50) | 0.02 |  | 1.88 (1.57-2.26) | < 0.001 |
| Model 5 | 1.04 (0.86-1.26) | 0.69 |  | 1.55 (1.29-1.86) | < 0.001 |

CI, confidence interval; HR, hazard ratio;

^1^ Model 1: univariable Cox regression model; Model 2: adjusted for sex and age; Model 3: included model 2 variables plus Townsend deprivation index, ethnic group, smoking status, diabetes, and cardiovascular disease ; Model 4: included model 3 variables plus systolic blood pressure, waist-to-hip ratio, total cholesterol, and high-density lipoprotein cholesterol; Model 5: included model 4 variables plus indicator variables for wearing glasses, having any hospital inpatient records, having any primary care clinical records, giving consent to touchscreen questionnaires.

#### Table S10. Reverse association analysis between age-related macular degeneration, glaucoma and the risk of sleep apnoea in CLSA

| **Model^1^** | **Glaucoma as exposure** | |  | **AMD as exposure** | |
| --- | --- | --- | --- | --- | --- |
|  | OR (95% CI) | P value |  | OR (95% CI) | P value |
| Model 1 | 1.06 (0.81-1.36) | 0.65 |  | 1.19 (0.90-1.54) | 0.21 |
| Model 2 | 1.08 (0.83-1.40) | 0.55 |  | 1.27 (0.95-1.66) | 0.09 |
| Model 3 | 1.06 (0.81-1.37) | 0.67 |  | 1.24 (0.93-1.62) | 0.13 |
| Model 4 | 1.05 (0.80-1.36) | 0.71 |  | 1.26 (0.95-1.65) | 0.10 |

AMD, age-related macular degeneration; CI, confidence interval; OR, odds ratio;

^1^ Model 1: univariable logistic regression model; Model 2: adjusted for sex and age; Model 3: included model 2 variables plus self-rated social standing, ethnic group, smoking status, diabetes, and heart disease; Model 4: included model 3 variables plus systolic blood pressure, waist-to-hip ratio, total cholesterol, and high-density lipoprotein cholesterol.

####

#### Table S11. Sensitivity analysis of the associations between sleep apnoea with the risk of age-related macular degeneration and glaucoma restricting to participants having hospital inpatient records in UK Biobank

| **Model^1^** | **Glaucoma event** | |  | **AMD event** | |
| --- | --- | --- | --- | --- | --- |
|  | HR (95% CI) | P value |  | HR (95% CI) | P value |
| Model 1 | 1.51 (1.26-1.82) | < 0.001 |  | 1.54 (1.27-1.87) | < 0.001 |
| Model 2 | 1.44 (1.19-1.73) | < 0.001 |  | 1.65 (1.36-2.00) | < 0.001 |
| Model 3 | 1.40 (1.16-1.69) | < 0.001 |  | 1.48 (1.22-1.80) | < 0.001 |
| Model 4 | 1.43 (1.18-1.72) | < 0.001 |  | 1.48 (1.22-1.79) | < 0.001 |
| Model 5 | 1.33 (1.11-1.61) | 0.003 |  | 1.39 (1.14-1.68) | < 0.001 |

AMD, age-related macular degeneration; CI, confidence interval; HR, hazard ratio;

^1^ Model 1: univariable Cox regression model; Model 2: adjusted for sex and age; Model 3: included model 2 variables plus Townsend deprivation index, ethnic group, smoking status, diabetes, and cardiovascular disease ; Model 4: included model 3 variables plus systolic blood pressure, waist-to-hip ratio, total cholesterol, and high-density lipoprotein cholesterol; Model 5: included model 4 variables plus indicator variables for wearing glasses, having any primary care clinical records, giving consent to touchscreen questionnaire.
